# Evidence of disease control: a realistic concept beyond NEDA in the treatment of multiple sclerosis

**DOI:** 10.12688/f1000research.11349.2

**Published:** 2017-06-20

**Authors:** Ana C. Londoño, Carlos A. Mora

**Affiliations:** 1 Instituto Neurológico de Colombia (INDEC), Medellín, Colombia; 2 Department of Neurology, MedStar Georgetown University Hospital, Washington, DC, USA

**Keywords:** axonopathy, biomarkers, MRI, MS, NEDA, neurodegeneration, neuroinflammation

## Abstract

Although no evidence of disease activity (NEDA) permits evaluation of response to treatment in the systematic follow-up of patients with multiple sclerosis (MS), its ability to accomplish detection of surreptitious activity of disease is limited, thus being unable to prevent patients from falling into a non-reversible progressive phase of disease. A protocol of evaluation based on the use of validated biomarkers that is conducted at an early stage of disease would permit the capture of abnormal neuroimmunological phenomena and lead towards intervention with modifying therapy before tissue damage has been reached.

## Introduction

Immunomodulatory therapies used in the treatment of patients with the clinical isolated syndrome (CIS) of multiple sclerosis (MS) and early relapsing remitting multiple sclerosis (RRMS), as well as the autologous hematopoietic stem cell transplant (aHSCT) used in the treatment of the catastrophic form of the disease, have accomplished a reduction in clinical relapses, a halting in progression toward neurological disability and have demonstrated a reduction of disease activity in MRI scans. This progress has led to the emergence of the ‘no evidence of disease activity’ (NEDA) composite which evaluates the response to these therapies in clinical studies, but its systematic application and utility in the clinical setting have not been established ^1^. NEDA could be considered not only a goal of therapy, but also as an indicator of prognosis and a tool that measures the effect of the medication currently being used ^2^. We propose a more aggressive approach that challenges the current application of NEDA.

## Fragility of the NEDA composite

In a cohort of 219 patients with either CIS or RRMS, 60 of 218 (27.5%) maintained NEDA status at 2 years, whereas only 17 of 216 (7.9%) had NEDA at 7 years. NEDA status at 2 years had a positive predictive value of 78.3% for no progression of disease at 7 years, demonstrating that it may be optimal in terms of prognostic value in the long term ^1^. This study disclosed a dissociation between clinical and MRI-followed disease activity, with a more prevalent loss of NEDA status determined by MRI changes at onset, followed by clinical relapses without presence of new lesions or changes in the previously existing lesions at later stages. The loss of NEDA status due to changes in expanded disability status scale (EDSS), which is a method of rating impairment in neurological functions excluding cognition, was infrequent in comparison to the determination provided by the clinical relapse and imaging-based biomarkers ^1^. These findings support a decrement in the inflammatory activity of disease as duration of disease increases, and the possibility of recruitment of additional neuronal pathways and/or a cortical remodeling that could compensate for the loss of function ^3^. Also, cognitive impairment may affect more than 82% of the patients with MS from early stages of the disease, affecting cognitive performance and quality of life ^3^. Damasceno *et al.* proposed that NEDA should also take into consideration other important measures of the patients’ neurological condition, such as their cognitive status and the volumetric analysis of the brain, converting NEDA into a completely effective tool for therapy evaluation. In their cohort of 42 patients with RRMS treated either with beta-interferon or glatiramer acetate, NEDA status was accomplished in only 30.8% of patients, with worsening of more than two cognitive domains in 58.3% of the NEDA group, and with evidence of cortical thinning and higher thalamus volume decrease in patients with MRI activity ^4^. Studies using drugs known to produce a better therapeutic effect in multiple sclerosis, such as natalizumab and alemtuzumab, have disclosed loss of the NEDA status after 2 years of initiation of therapy in 37% and 39% of the treated patients, respectively ^4^. Currently, aHSCT is the only therapeutic approach that has accomplished NEDA status after 3 years in 75% of patients ^5^. Giovannoni has recently discussed the adaptation of NEDA to the type of the therapeutic regime, and has considered three scenarios. These include a) no treatment, b) maintenance/escalation of disease-modifying therapy, and c) use of induction therapies (such as alemtuzumab, cladribine and aHSCT) establishing a baseline according to the pharmacodynamics of each drug available ^2^.

Although progression of disease, which reflects the neurodegenerative component of MS, is expected to be reflected by the EDSS score in the actual concept of NEDA, evidence has shown that the T25FW (timed 25-foot walk) test gave better documentation of clinical progression ^1^. Considering the use of NEDA, Dadalti Fragoso discussed the difficulties encountered when using EDSS to objectively document patient functionality in different areas. Clinical manifestations such as fatigue or sensitivity to heat are not considered, there is an inconvenient variability among evaluators with differences up to 2.0 points for EDSS and 3.0 points for functional system evaluation, and there is the disadvantage of having the patient, and not the evaluator, reporting the ability to walk 500 m or 300 meters ^6^. These studies have shown that, in a high percentage of patients, activity of disease was present at baseline and could not be detected by NEDA thus resulting in a delayed therapeutic intervention and irreparable damage of the central nervous system (CNS).

## Background activity beyond the surveillance of NEDA

The term ‘minimal evident disease activity’ (MEDA) has been applied to MS patients who have been apparently stable in comparison to patients with higher level of activity in the short to intermediate term ^2^. Thus, beyond documenting NEDA we mostly need to achieve documentation of the unnoticed surreptitious activity of disease which remains despite treatment. The determination of biomarkers of inflammation and neurodegeneration in body fluids, combined with the use of non-conventional MRI with the ability to detect changes in the normal appearing brain tissue, could assist in the detection of a sequence of cellular and molecular events, inside and out of the CNS, that occur before fulfilling the current definition of NEDA composite. Multiple biomarkers have been identified in MS, but their validation and clinical application have not been established ^7, 8^. Teunissen recently discussed the use of biomarkers for MS such as N-acetylaspartate (representing mitochondrial dysfunction and/or neuro-axonal loss), chitinase 3 like-protein 1 (meaning reactive astrogliosis and microglial activity), neurofilament light chain (related to axonal loss) and glial fibrillary acidic protein (representing astrocytic cytoskeleton injury). They have been validated in at least two independent cohorts, and evaluation of their expression could be a useful tool at the time of diagnosis of MS, and during follow-up after the administration of disease modifying therapy ^9^.

Bonnan *et al.* have recently suggested that NEDA cannot predict sustained remission or complete recovery of disease, and proposed a ‘disease free status score’ be established, based on whether or not there is biological activity of disease, by measuring the level of biomarkers in CSF ^10^. Taking into consideration that, at present, there are no therapeutic agents available that would be able to offer a cure for MS, a disease free status score could create confusion on top of becoming a non-realistic concept. We support the concept that the methodology used to determine the stage of disease should be based on the measuring of the level of biomarkers involved in the inflammatory and neurodegenerative events of disease, not only by CSF analysis but also with non-invasive available tools such as PET-CT and non-conventional MRI to evaluate the normal appearing brain tissue ^11– 16^.

## Goals and conclusion

In the systematic evaluation of the patient with MS, the primary goal should be the monitoring of evidence of disease control with biomarkers in order to:

- 1. Prevent clinical relapses
- 2. Confirm absence of changes suggestive of progression of the disease in pre-existing lesions, including checking for presence of new lesions or atrophy detected by MRI; and
- 3. Prevent progression toward disability

By supporting a pro-active management of disease, avoiding brain tissue injury instead of controlling existing inflammation and/or neurodegeneration, this approach is promoting a personalized management of disease ^17^.

Early treatment of patients with CIS has led to the identification of a therapeutic window that, with current interventions, would be able to slow down progression toward higher scores in EDSS evaluation ^3^. Taking into consideration the recent significant attention given to novel monoclonal antibody therapies (including alemtuzumab, rituximab, ocrelizumab, daclizumab) ^18^– ^21^ and stem cell therapies (aHSCT) ^22^, our most immediate goal should involve searching for strategic interventions to control both inflammation and neurodegeneration, hopefully reaching a stage of prolonged remission in selected patients. Taking into consideration that NEDA status is currently able to switch on red flags only when tissue damage has already occurred in the CNS, we believe that ‘evidence of disease control’ will be accomplished through a better defined, convincing and more realistic monitoring of validated biomarkers.

The current clinical-pathological method of analysis of diseases is evolving toward a holistic approach that characterizes human disease focused on the total integration of its composing parts (genetics, genomics, biochemical, cellular informatics, physiology, clinics, etc) the interactions of which are multiple, dynamic and interdependent ^23^. These components include a group of networks, the disruption of which will furnish relevant biological information able to transform disease classification, not from phenotype but from molecular presentation, allowing preclinical diagnosis, and individualizing therapy ^24^. The study of MS pathogenesis from the perspective of biological systems interaction will permit the understanding of its complexity [see Table 1] ^23^. Thus, ongoing characterization of biomarkers in the study of patients with MS is becoming an assertive step in the right direction.

**Table 1.** From clinical assessment to application of systems biology in MS.

Networks	BASED ON
**NEDA status** ^25^ • NEDA 1–3 • NEDA 4 • NEDA 5 • NEDA 6 • NEDA 7 • NEDA 8	Clinical event, EDSS, MRI Brain atrophy Cognitive impairment CSF neurofilament level Patients related outcome Oligoclonal bands
**BIOMARKERS** ^7, 8^ Related to cells, structures, metabolic pathways, and inflammatory and degenerative cascades.	OCB, NAA, Glutamate, IL-12, IL-23, Enolase, NfL, CXCL13, IL-8, ATP break down products, GFAP, MMP9, MMP3, Th17, Th1/Th17, Chitinase 3, IFN-ϒ, TNF-α, Fetuin-A, Osteopontin, PET (microglia), non-conventional MRI. Others.
**NEURO-IMMUNO-PATHOLOGY** 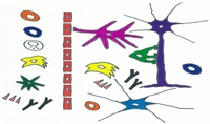	Tissue damage resulting from interaction of neurons, glial cells, immune system cells (B & T-cell lymphocytes, macrophages, dendritic cells), cytokines and antibodies in the CNS
**GENETIC DETERMINANTS** ^8^ 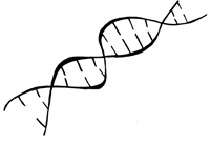	HLA-DRB1*1501: • risk for MS • early disease onset. • risk for progression from RRMS to SPMS HLA-DPB1* 0501 & HLA-DPB1* 0301: • risk for opticospinal MS. HLA-DRB1* 1501 & HLA-DQB1* 0301: • worst brain atrophy measures. DR3 & DR4 haplotypes: • risk for MS. TOB1 gen: • early conversion from CIS to CDMS. ApoEƐ4: • greater risk for mental disorders. Pharmacogenomics
**GUT-IMMUNE-BRAIN AXIS** ^26^ The gut-associated lymphoid tissue system (GALT) is located in the intestinal lamina propria, near the epithelium, and consists of: Organized cellular complexes • Peyer’s patches • solitary lymphoid follicles Dispersed cells • T and B cells, macrophages, and dendritic cells	The human intestinal flora (microbiota) interaction with GALT could be considered harmless, beneficial or pathogenic depending on the anti-inflammatory or pro-inflammatory state that could result from this interaction. In the later, it could influence immunity and inflammation in the pathophysiology of MS.

The table shows the dynamic interaction of networks leading to the molecular expression of MS in the preclinical state and through disease span. **Abbreviations in the table: EDSS**: Expanded Disability Status Scale; **OCB**: oligoclonal bands; **NAA**: N-Acetyl aspartate; **IL**: interleukin; **NfL**: light chain sub-unit of neurofilaments; **CXCL13**: C-X-C Motif Chemokine Ligand 13; **ATP**: adenosine triphosphate; **GFAP**: glial fibrillary acidic protein; **MMP**: matrix metalloproteinase; **Th17**: IL-17 secreting T-lymphocyte; **IFN**-ϒ: interferon gamma; **TNFα**: tumor necrosis factor α; **CIS**: clinical isolated syndrome; **CDMS**: clinical definitive multiple sclerosis
