# Supplementary material for: Evidence of disease control: a realistic concept beyond NEDA in the treatment of multiple sclerosis
Source: F1000Res. 2017 Jun 20;6:566. Originally published 2017 Apr 25. [Version 2] doi: 10.12688/f1000research.11349.2 (PMC5446020; doi:10.12688/f1000research.11349.2)
Supplement: Peer review history

# Peer review: Referee response for version 1

**DOI:** 10.5256/f1000research.12251.r22238

**Published:** 2017-05-24

**Authors:** Declan Chard

**Affiliations:** 1 Institute of Neurology, University College London, London, WC1N 3BG, UK

With a view to preventing disability by optimising treatment decisions, there has been growing interest in the concept of ‘no evidence of disease activity’ (NEDA) outcomes. In their opinion article Londoño and Moro consider the complexities of NEDA definitions and the role of such scores in clinical practice.

 They authors remind us that the definition of NEDA as a marker of treatment failure and predictor of future clinical outcomes is subject to debate. While considering definitions of NEDA, it is perhaps also worth mentioning the work of Rio and colleagues (Multiple Sclerosis. 2009 Jul;15(7):848–53.), which suggests that radiological evidence of disease activity does not necessarily translate into a significantly increased short-term risk of clinical disease activity.

 The authors consider the limitations of using EDSS scores as a marker of clinical progression, to which could perhaps be added the difficulties determining if an episode of symptoms is due to inflammation (Tallantyre *et al. *2015 ^1^) They also highlight that MRI measures of lesion accrual or brain atrophy may still overlook clinically relevant disease activity, and that fluid biomarkers may also provide relevant indicators of evolving pathology.

 On reviewing the work of Rotstein *et al.* (2015) ^2^ the authors note that ‘loss of NEDA status due to changes in expanded disability status scale (EDSS), which is a method of rating impairment in neurological functions excluding cognition, was infrequent.’ While less frequent than relapses, the figures for people with established MS shown in Table 2 (if I have read these correctly) suggest that by 7 years about 20% of those with evidence of clinical activity had progression without relapses (74% had evidence of either progression or relapse, 59% had had a relapse, implying that 15% had progression without a relapse).

 The authors ‘support the concept that the methodology used to determine the stage of disease should be based on the measuring of the level of biomarkers involved in the inflammatory and neurodegenerative events of disease’. This touches on an interesting line of thought on differentiating MS subtypes, which clinically can be difficult (and I am not aware of any biomarkers that substantially improve on this on a person-by-person basis), and how this relates to definitions of NEDA or ongoing clinically relevant disease activity. With regard to clinical outcomes, while NEDA definitions include both relapses and disability progression, the two may not be closely linked (Vukusic and Confavreux 2007 ^3^). As such, predictors of the risk of future relapses and risk of non-relapse associated progression may differ, and similarly composite scores designed to predict these outcomes may not necessarily be the same, or applicable to all MS subtypes equally. It would be interesting to hear more of the authors’ thoughts on this.

 In the abstract, the authors state that ‘A protocol of evaluation based on the use of validated biomarkers that is conducted at an early stage of disease would permit the capture of abnormal neuroimmunological phenomena and lead towards intervention with modifying therapy before tissue damage has been reached.’ However, there are perhaps some qualifications to this if a biomarker protocol is going to be useful in clinical practice, for example that the biomarkers used need to be (alone or in combination) reliable markers of disease activity at the level of individual people with MS, and that the pathological processes they reflect can be effectively targeted by treatments.

I have read this submission. I believe that I have an appropriate level of expertise to confirm that it is of an acceptable scientific standard.

## References

[1] TallantyreECCausonEGHardingKEPickersgillTPRobertsonNP: The aetiology of acute neurological decline in multiple sclerosis: experience from an open-access clinic. *Mult Scler*.2015;21(1) : 10.1177/1352458514538333 67-75 10.1177/1352458514538333 24948684

[2] RotsteinDLHealyBCMalikMTChitnisTWeinerHL: Evaluation of no evidence of disease activity in a 7-year longitudinal multiple sclerosis cohort. *JAMA Neurol*.2015;72(2) : 10.1001/jamaneurol.2014.3537 152-8 10.1001/jamaneurol.2014.3537 25531931

[3] VukusicSConfavreuxC: Natural history of multiple sclerosis: risk factors and prognostic indicators. *Curr Opin Neurol*.2007;20(3) : 10.1097/WCO.0b013e32812583ad 269-74 10.1097/WCO.0b013e32812583ad 17495619

# Response

**Published:** 2017-06-12

**Authors:** Carlos Mora

**Affiliations:** Georgetown University Hospital, Washington DC, USA

We strongly appreciate the comments and input provided by the reviewers of the first version of our article. With especial interest in the comments and questions posed by reviewer 2 (Dr Chard) we acknowledge the work of Rio and colleagues (Multiple Sclerosis. 2009 Jul;15(7):848-53) and we take into consideration the fact that although the mentioned study was limited to the effect of one disease modifying agent (IFNb given in four different commercially available preparations) on patients with the relapsing-remitting form of disease [RRMS] it showed that progression in at least two of the three variables analyzed (relapses, increase of disability or MRI activity) after 12 months would correlate with risk of progression of disease in the following years. However, this conclusion cannot be extrapolated to other groups of patients with RRMS who have happened to be treated with other disease modifying agents taking into consideration the different mode of action of these immune-modulators. In relation to the comment on our citation of Rotstein and colleagues (JAMA Neurol. 2015;72(2):152–158. 25531931 10.1001/jamaneurol.2014.3537) we extended the content of the sentence in the text reflecting that the loss of NEDA status due to changes in EDSS was infrequent in comparison to the level of determination provided by the clinical relapsing and imaging-related biomarkers.

As for the invitation to comment on how the different MS sub-types may correlate to definitions of NEDA or ongoing clinically relevant disease activity we concur with the fact that although NEDA definitions include both relapses and disability progression, the two may not be closely linked and, therefore, a different set of biomarkers ought to be validated to determine forthcoming inflammation and/or neurodegeneration. On the other hand, differentiation of sub-types of disease (especially in reference to inflammatory vs. degenerative pathologic processes) will be feasible with the forthcoming application of biomarkers in the MS related clinical practice. NEDA may not be a useful tool for the evaluation of patients with PPMS taking into consideration that this sub-type of MS is characterized by on-going disability and minimal inflammatory activity in MRI. At present, with the development of new medications for the treatment of PPMS, it would be essential to count with measurable biomarkers which would allow the determination of an objective response to therapies.

# Peer review: Referee response for version 1

**DOI:** 10.5256/f1000research.12251.r22236

**Published:** 2017-05-02

**Authors:** Hans Lassmann

**Affiliations:** 1 Center for Brain Research, Medical University of Vienna, Vienna, Austria

This opinion article deals with a highly relevant topic, the validity of the concept of no evidence for disease activity "NEDA" in multiple sclerosis.  Prevention of disease progression may possibly be achieved in patients, when treatment completely blocks ongoing disease activity and NEDA has been suggested as a tool to check, whether this is achieved in individual patients. However, clinical detection of disease activity, as suggested in the NEDA concept is far from being complete. Thus, this opinion article suggests to supplement current NEDA criteria with additional para-clinical markers. Such potential biomarkers are non-conventional new MRI sequences, magnetic resonance spectroscopy (N-acetyl aspartate) for evaluation of mitochondrial injury and biochemical markers such as chitinase 3, neurofilament or glial fibrillary acidic protein in the cerebrospinal fluid.  Whether these additional markers will increase the reliability of NEDA criteria, will have to be established in prospective clinical studies. However, so far none of them are perfect para-clinical predictors of disease activity and currently no tools are available to monitor major pathological substrates of disease progression in MS, such as for instance the dynamic development of demyelinating lesions in the cortex, the expansion of pre-existing white matter lesions or the presence of more subtle changes within the normal appearing white and grey matter of the MS brain.

I have read this submission. I believe that I have an appropriate level of expertise to confirm that it is of an acceptable scientific standard.
